# A behavioral approach to shared mapping of peripersonal space between oneself and others

**DOI:** 10.1038/s41598-018-23815-3

**Published:** 2018-04-03

**Authors:** Wataru Teramoto

**Affiliations:** Department of Psychology, Kumamoto University, 2-40-1 Kurokami, Chuoku, Kumamoto, 860-8555 Japan

## Abstract

Recent physiological studies have showed that some visuotactile brain areas respond to other’s peripersonal spaces (PPS) as they would their own. This study investigates this PPS remapping phenomenon in terms of human behavior. Participants placed their left hands on a tabletop screen where visual stimuli were projected. A vibrotactile stimulator was attached to the tip of their index finger. While a white disk approached or receded from the hand in the participant’s near or far space, the participant was instructed to quickly detect a target (vibrotactile stimulation, change in the moving disk’s color or both). When performing this task alone, the participants exhibited shorter detection times when the disk approached the hand in their near space. In contrast, when performing the task with a partner across the table, the participants exhibited shorter detection times both when the disk approached their own hand in their near space and when it approached the partner’s hand in the partner’s near space but the participants’ far space. This phenomenon was also observed when the body parts from which the visual stimuli approached/receded differed between the participant and partner. These results suggest that humans can share PPS representations and/or body-derived attention/arousal mechanisms with others.

## Introduction

Humans interact with each other in daily life. Especially when working with others, one needs to understand the actions, goals, and situations, including sensory experiences, of others in order to optimize his/her own actions. One way to realize these functions is to remap others’ actions and bodies onto one’s own representations in the brain, as can be done with mirror neurons^1–3^. The mirror neurons are mainly found in premotor and parietal brain areas that are activated during the monkey’s own actions and when the monkey observes another monkey performing the corresponding actions. Because these neurons directly match the visual images of other’s actions with the representation of his/her own actions, they are thought to be involved in social cognitive functions, such as recognizing and imitating actions of others^4,5^. Recent neuroimaging studies have reported that some human somatosensory cortex neurons are activated both when ones’ own body is touched and when one observes another individual being touched on the corresponding body part^6–10^. These neural substrates are probably involved in behaviors such as automatic mimicry, which involves subconscious imitation of the expressions of others^11^, and visual remapping of touch, such as when tactile sensitivity on one’s face is enhanced by viewing another person’s face being touched^12,13^. These findings suggest that the brain interprets other’s actions and perceptions by remapping them onto one’s own bodily representations.

More recent studies have shown that representations of the space immediately around body parts, called peripersonal space (PPS)^14,15^, are also shared between oneself and others^16–18^. Because most of the events and objects that we directly interact with are found immediately around our bodies, this space is considered pivotal for eliciting goal-directed and defensive behaviors in connection with external objects^19–22^. In fact, electrophysiological and neuroimaging studies have reported that this space is represented by visuotactile and audiotactile neurons in the premotor and parietal cortices^15,23–28^. Ishida *et al*.^16^ showed that some visuotactile neurons in the ventral intraparietal area responded both when a monkey’s own body is touched and when the corresponding body part of an experimenter facing the recorded monkey is touched. Furthermore, they found visuotactile neurons that exhibit similar responses to visual stimuli presented near a monkey’s own body parts and to those presented near the experimenter facing the recorded monkey. In humans, Brozzoli *et al*.^17^ used neuroimaging techniques to detect the same visual stimulus response pattern in neural populations in the premotor cortex. These findings suggest that the brain has mechanisms to automatically remap another individual’s PPS onto self-representations, but little is known about how this affects human behavior.

Maister *et al*.^18^ conducted the only published behavioral investigation into this remapping phenomenon. They asked their participants to report tactile stimuli as quickly as possible while listening to an approaching sound^29^. When a participant performed this audiotactile interaction task alone, the auditory stimulus facilitated tactile detection when it was located close to the participant’s body. When participants performed the task with partners seated in front of, but far from them, the auditory stimulus facilitated tactile detection when it was located close to the partner’s body as well. This may be behavioral evidence for the remapping of other’s PPS onto one’s own PPS representation. Importantly, this phenomenon did not occur until the participants had built feelings of ownership over their partner’s body through shared sensory experiences eliciting an enfacement illusion^30^. This is an important difference from earlier neurophysiological and neuroimaging studies^16,17^, which did not induce such ownership illusions.

**Figure 1:**
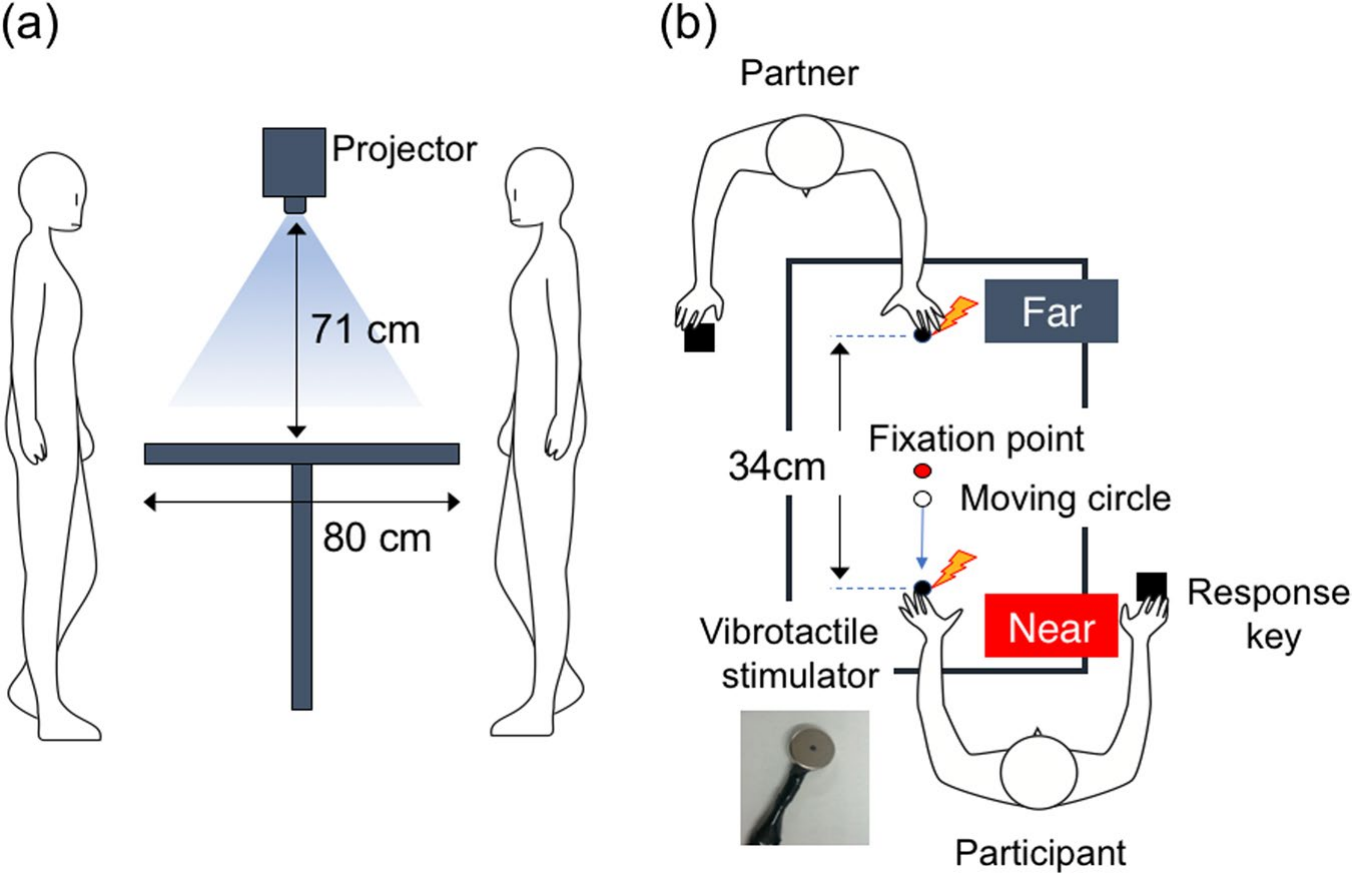
Schematic illustrations of the experimental setup (**a**) and stimuli (**b**) used in this study. (**a**) Setup: Visual stimuli were projected onto a tabletop screen via a projector. To increase the visibility of visual stimuli in the far zone, the participants stood in front of the table while watching the screen. The participants touched their heads to an apparatus connected to the projector in order to keep the viewing distance constant. When the participants performed the task with the partner, the partner stood across the table while facing the participants and touching his forehead to the opposite side of the apparatus. The experiment room was dimly lit, but the projector’s light illuminated the partner’s hand for the participants. (**b**) Stimuli: Each participant placed his or her left hand palm down on a table at a predetermined position, and a vibrotactile stimulator with a 0.95-cm diameter was attached to the tip of the index finger. A red fixation point with a 0.8-cm diameter was presented at the table’s center on a black background. The fixation point was located 17 cm from the tip of the index finger. A disk with a 1.2-cm diameter approached or receded from the participant’s hand at 9 cm/s for 1 s in either the area beyond the fixation point (the far space) or the area nearer the participant (the near space). When the disk moved in the near space, the very end of the fingertip was where the approaching disk vanished and the receding disk appeared. When the disk moved in the far space, the corresponding point was 8 cm beyond the fixation point.

**Figure 2:**
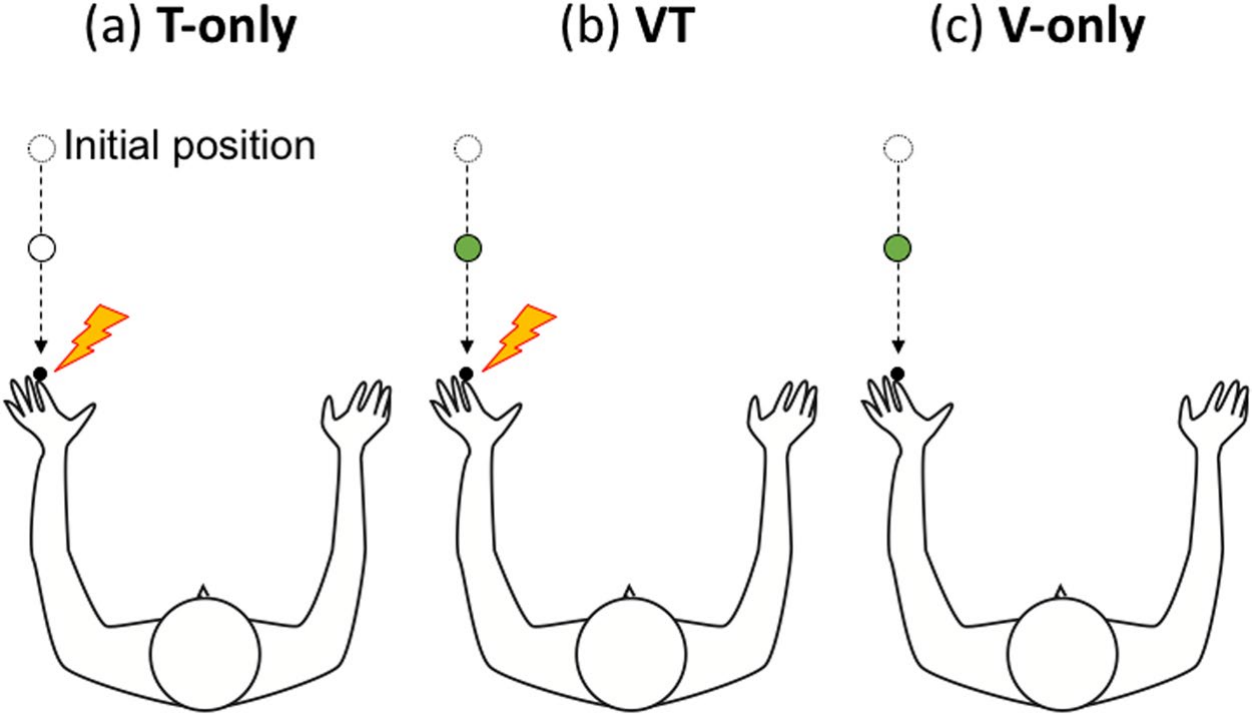
Schematic illustrations of target types: T-only (**a**), VT (**b**), and V-only (**c**). Only targets approaching in the near space are shown. (**a**) Under the T-only condition, a vibrotactile stimulation was presented during the disk’s movement. (**b**) Under the VT condition, both vibrotactile stimulation and a change in the moving disk’s color were presented during the disk’s movement. The disk was initially white and turned green. (**c**) Under the V-only condition, only a change in the moving disk’s color was presented during the disk’s movement. Each target type was presented in 30% of trials, and the remaining 10% were catch trials featuring no target presentation. The target appeared 0.2, 0.5, or 0.8 s after the disk started moving.

I conducted this study to rigorously test for behavioral signs of PPS remapping without such prior induction of ownership illusions. The remapping phenomenon implies that the responses when stimuli approach participants should be the same as those when stimuli recede from the participants but approach the partner, so I tested both approaching and receding conditions. Additionally, as in previous neurophysiological and neuroimaging studies, I focused on visuotactile interactions^16,17^ instead of audiotactile ones. While a disk was either approaching or receding from the participants’ hands in either far or near zones (Fig. 1), the participants were asked to respond to visual (V-only condition), tactile (T-only condition), or visuotactile targets (VT condition) as soon as possible (Fig. 2). Previous electrophysiological studies reported that body-approaching visual stimuli in the near space evoked greater activity in visuotactile neurons than those in the far space^26^. I therefore expected that when the participants performed the task without a partner (single condition), tactile targets would be detected faster when the disk approached the hand in the near space than when the disk receded from the hand or moved in the far space. If the partner’s presence (paired condition) did not affect target detection, then the same results would be obtained under both the single and paired conditions. However, if the partner’s PPS was remapped onto the participant’s self-representations, then tactile detection would be facilitated both when the disk approached the participant’s hand in the near space and when the disk receded from the participant’s hand but approached the partner’s hand in the partner’s near space (i.e., the participant’s far space). I obtained clear behavioral evidence that the participants behaved as if the partner’s near space were their own near space, without the induction of ownership illusions.

## Results

Each participant placed his or her left hand palm down on a table at a predetermined position, and a small vibrotactile stimulator was attached to the tip of the index finger. Visual stimuli were projected onto a tabletop screen via a projector. The participants reported the presence of a target by pressing a response button with their right hands, irrespective of the disk’s spatial distance (near or far), trajectory (approaching or receding), and target type (T-only, V-only, or VT). These conditions were randomly presented in each session. The single and paired conditions trials were conducted in different sessions.

**Figure 3:**
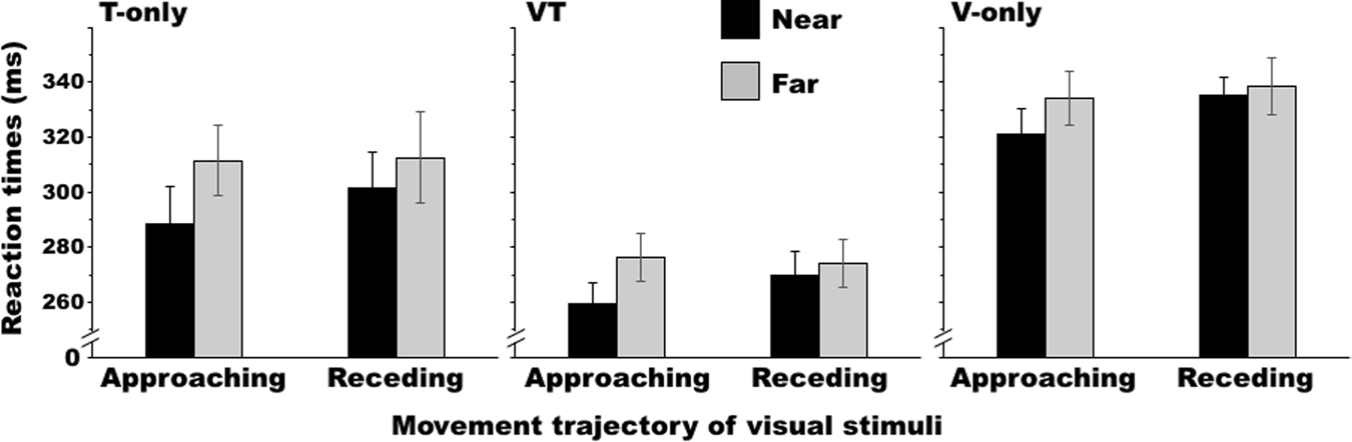
Average Experiment 1 reaction times across the participants (n = 10) for each target type when performing the task alone (single condition). Error bars show standard errors of the means.

Trials in which the reaction times (RTs) exceeded ± 2 standard deviations or the lower limit (150 ms) were discarded from the following analyses as errors. In Experiment 1, under the single condition, the error rates, including no-response errors, were 5.3 ± 6.9% overall and 5.6 ± 8.0%, 4.4 ± 6.4%, and 5.8 ± 6.3% under the T-only, VT, and V-only conditions, respectively. Most errors arose from premature responses. The false alarm rate for catch trials was 13.2 ± 16.3%. Figure 3 shows the average RTs across participants. Two-way repeated analyses of variance (ANOVAs) with within-participant factors of target distance and trajectory for each target type revealed significant or nearly significant main effects of distance for all target types (T: *F*_1,9_ = 42.27, *p* < 0.001, *η*_*G*_^2^ = 0.041; VT: *F*_1,9_ = 11.21, *p* = 0.009, *η*_*G*_^2^ = 0.040; V: *F*_1,9_ = 4.11, *p* = 0.073, *η*_*G*_^2^ = 0.028) and trajectories (T: *F*_1,9_ = 4.79, *p* = 0.056, *η*_*G*_^2^ = 0.013; VT: *F*_1,9_ = 4.10, *p* = 0.073, *η*_*G*_^2^ = 0.006; V: *F*_1,9_ = 10.16, *p* = 0.011, *η*_*G*_^2^ = 0.021). The RTs were shorter when the disk was located in the near zone than in the far zone and also shorter when the disk was approaching than when it was receding. The ANOVAs also identified a nearly significant interaction only for the VT condition (T: *F*_1,9_ = 2.25, *p* = 0.168, *η*_*G*_^2^ = 0.025; VT: *F*_1,9_ = 3.95, *p* = 0.078, *η*_*G*_^2^ = 0.015; V: *F*_1,9_ = 2.85, *p* = 0.126, *η*_*G*_^2^ = 0.009) and further analysis of this interaction revealed that RTs for the near condition were shorter than those for the far condition only when approaching disks were presented (approaching: *F*_1,18_ = 14.20, *p* = 0.001, *η*_*G*_^2^ = 0.100; receding: *F*_1,18_ = 0.90, *p* = 0.355, *η*_*G*_^2^ = 0.006). In summary, the participants detected targets sooner in single condition trials when the disk was in the near space than when it was moving in the far space and for the VT condition, this was only salient when the disk was approaching the participants’ hands.

**Figure 4:**
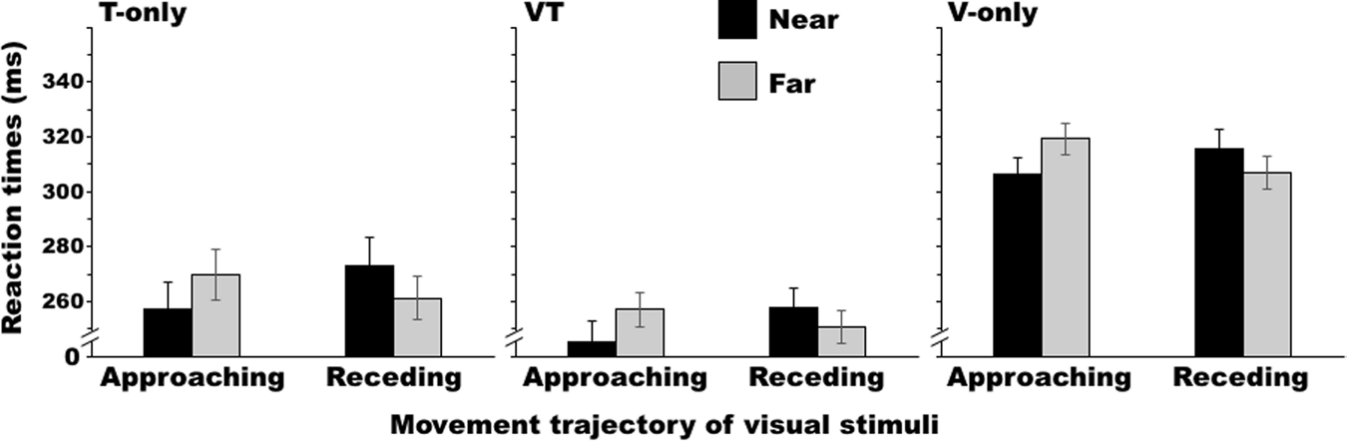
Average Experiment 1 reaction times across the participants (n = 10) for each target type when performing the task with a partner (paired condition). Error bars show standard errors of the means.

Under the paired condition, the error rates were 4.0 ± 5.2% overall and 4.4 ± 5.0%, 3.9 ± 5.8%, and 3.8 ± 4.9% for the T-only, VT, and V-only conditions, respectively. Most errors arose from premature responses. The false alarm rate for catch trials was 18.1 ± 12.9%. Figure 4 shows the average RTs across participants. The ANOVAs revealed a significant interaction between distance and trajectory for all target types (T-only: *F*_1,9_ = 19.78, *p* = 0.002, *η*_*G*_^2^ = 0.048; VT: *F*_1,9_ = 17.55, *p* = 0.002, *η*_*G*_^2^ = 0.050; V-only: *F*_1,9_ = 13.72, *p* = 0.005, *η*_*G*_^2^ = 0.079). Further analysis of this interaction revealed that the RTs were significantly shorter when the disk approached in the near zone than when it approached in the far zone (T-only: *F*_1,18_ = 9.95, *p* = 0.006, *η*_*G*_^2^ = 0.047; VT: *F*_1,18_ = 15.68, *p* = 0.001, *η*_*G*_^2^ = 0.071; V-only: *F*_1,18_ = 15.61, *p* = 0.001, *η*_*G*_^2^ = 0.125), but this relationship was reversed for receding disks (T-only: *F*_1,18_ = 9.54, *p* = 0.006, *η*_*G*_^2^ = 0.049; VT: *F*_1,18_ = 5.55, *p* = 0.030, *η*_*G*_^2^ = 0.030; V-only: *F*_1,18_ = 5.71, *p* = 0.028, *η*_*G*_^2^ = 0.044). Target detection was thus facilitated both when the disk approached the participants’ hands and when it approached the partner’s hand (I conveniently call this phenomenon the “remapping effect”).

It should be noted that the RTs were shorter under the paired condition than under the single condition. This cannot be an effect of condition presentation order because the presentation order was counterbalanced across participants. Rather, it probably results from social facilitation, in which performing a task with a partner improves outcomes^31^. Several studies have confirmed that this occurs in RT tasks^32,33^.

Regarding my observation of remapping effects without prior induction of body ownership illusions, which is inconsistent with Maister *et al*.’s^18^ results, it is possible that the ongoing shared sensory experience between the participants and the partners contingently induced body ownership illusions and consequent remapping because the participants and the partners responded to all stimuli together. I investigated this possibility by testing the effect of session using data from the main experiment. Because each participant completed three paired condition sessions, the remapping effect should have been stronger in later sessions if body ownership illusions were involved. However, no effect of session was observed under any modality condition (Supplementary Fig. S1).

One question is whether the acquaintanceship between my participants and their partner could be crucial for the remapping effect. My participants were acquainted with their partner for years. Maister *et al*.^18^ speculated that shared activities in daily life might facilitate remapping of another individual’s PPS onto one’s own PPS representations. Previous studies showed that acquaintances tend to mimic each other’s movements and postures^34,35^, which lends credibility to this possibility. Another question is whether the presence of another person was required. Studies have reported that mere exposure to dummy hands can influence one’s own positional sense and PPS representations^28,36^, while previous neurophysiological and neuroimaging studies investigating PPS remapping showed that remapping was weak when rubber hands were presented instead of real ones^16,17^. To answer these questions, I recruited new participants and a partner who had never met the participants and conducted Experiment 2. In addition to the single and paired conditions, I included a rubber hand condition, in which a left forearm rubber hand with a vibrator attached to the tip of the index finger was placed on the table instead of the partner and his real hand. These conditions were conducted in different sessions.

**Figure 5:**
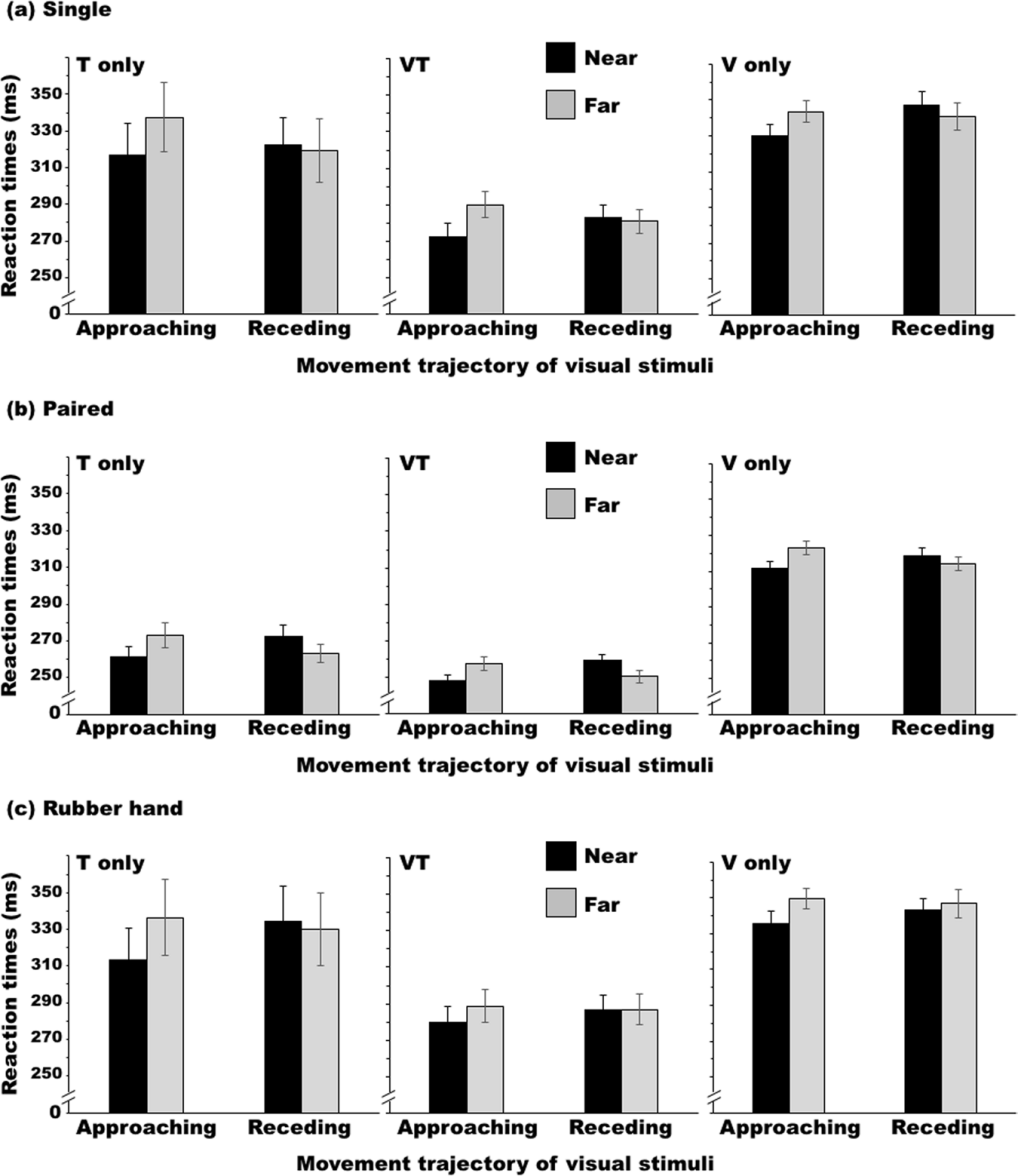
Average Experiment 2 reaction times (RTs) across the participants (n = 11) under the (**a**) single condition, (**b**) paired condition, and (**c**) rubber hand condition. Error bars show standard errors of the means.

Figure 5 shows the results of Experiment 2. The error rates, including no-response errors, were 1.5 ± 1.0% for the single condition, 6.5 ± 5.5% for the paired condition, and 2.4 ± 2.2% for the rubber hand condition. Two-way ANOVAs were applied to the RT data for each target condition with the target distance and trajectory as within-participant factors of target distance and trajectory. Under the single condition, the ANOVA revealed a significant interaction for all target types (*Fs*_1,10_ > 15.62, *ps* < 0.003, *η*_*G*_^2^*s* > 0.012). Further analysis of this interaction revealed that the RTs were shorter when the disk approached in the near space than when it approached in the far space (*Fs*_1,20_ > 11.61, *ps* < 0.003, *η*_*G*_^2^*s* > 0.031) but that no significant effect of distance emerged when the disk receded from the hand (*Fs*_1,20_ < 2.78, *ps* > 0.111, *η*_*G*_^2^*s* < 0.019). Under the paired condition, the ANOVA identified a significant interaction for all target types (*Fs*_1,10_ > 8.94, *ps* < 0.014, *η*_*G*_^2^*s* > 0.092). Analyses of this interaction revealed that the RTs were shorter when the disk approached in the near space than when it approached in the far space (*Fs*_1,20_ > 6.53, *ps* < 0.019, *η*_*G*_^2^*s* > 0.080) but that this relationship was reversed when the disk receded from the hand under the T-only and VT conditions (T-only: *F*_1,20_ = 5.89, *p* = 0.025, *η*_*G*_^2^ = 0.060; VT: *F*_1,20_ = 16.09, *p* < 0.001, *η*_*G*_^2^ = 0.164; V-only: *F*_1,20_ = 0.97, *p* = 0.337, *η*_*G*_^2^ = 0.027). For the rubber hand condition, a significant or nearly significant interaction was also observed for the T-only and VT conditions (T-only: *F*_1,10_ = 11.04, *p* = 0.008, *η*_*G*_^2^ = 0.012; VT: *F*_1,10_ = 4.36, *p* = 0.064, *η*_*G*_^2^ = 0.007; V-only: *F*_1,10_ = 2.79, *p* = 0.126, *η*_*G*_^2^ = 0.014). Analyses of this interaction revealed that the RTs were shorter when the disk approached in the near space than when it approached in the far space (T-only: *F*_1,20_ = 21.86, *p* < 0.001, *η*_*G*_^2^ = 0.035; VT: *F*_1,20_ = 6.81, *p* = 0.017, *η*_*G*_^2^ = 0.025) but that no significant effect of distance emerged when the disk receded from the hand (T-only: *F*_1,20_ = 0.73, *p* = 0.402, *η*_*G*_^2^ = 0.001; VT: *F*_1,20_ = 0.00, *p* = 0.986, *η*_*G*_^2^ < 0.001). For the V-only condition, a significant main effect was only observed for distance, with faster responses being observed for motion in the near space than in the far space (*F*_1,10_ = 4.97, *p* = 0.049, *η*_*G*_^2^ = 0.040). In summary, under the paired condition, the remapping effects were observed for the T-only and VT conditions but not for the V-only condition. There was no remapping effect under the single and rubber hand conditions. These results suggest that acquaintanceship is not a critical factor, although it may modulate the remapping effect, and that dummy hands are insufficient for inducing the remapping effects.

In the physiological literature, it is well-known that PPS is represented in a body part-specific manner^37,38^. Ishida *et al*.^16^ showed that shared mapping of PPS around the face occurred when the visual stimulus was presented near the partner’s face but not when it was presented near the partner’s chest. To investigate whether the phenomenon found in my previous experiments was similar to those that have been reported in monkeys, I recruited 18 participants and conducted an additional experiment (Experiment 3). Two conditions were included in this experiment: a single condition, which was the same as those in the previous experiments, and a paired-incongruent condition, whereby the body parts that were approached or receded by the visual stimuli differed between the participant (hand) and partner (chest).

**Figure 6:**
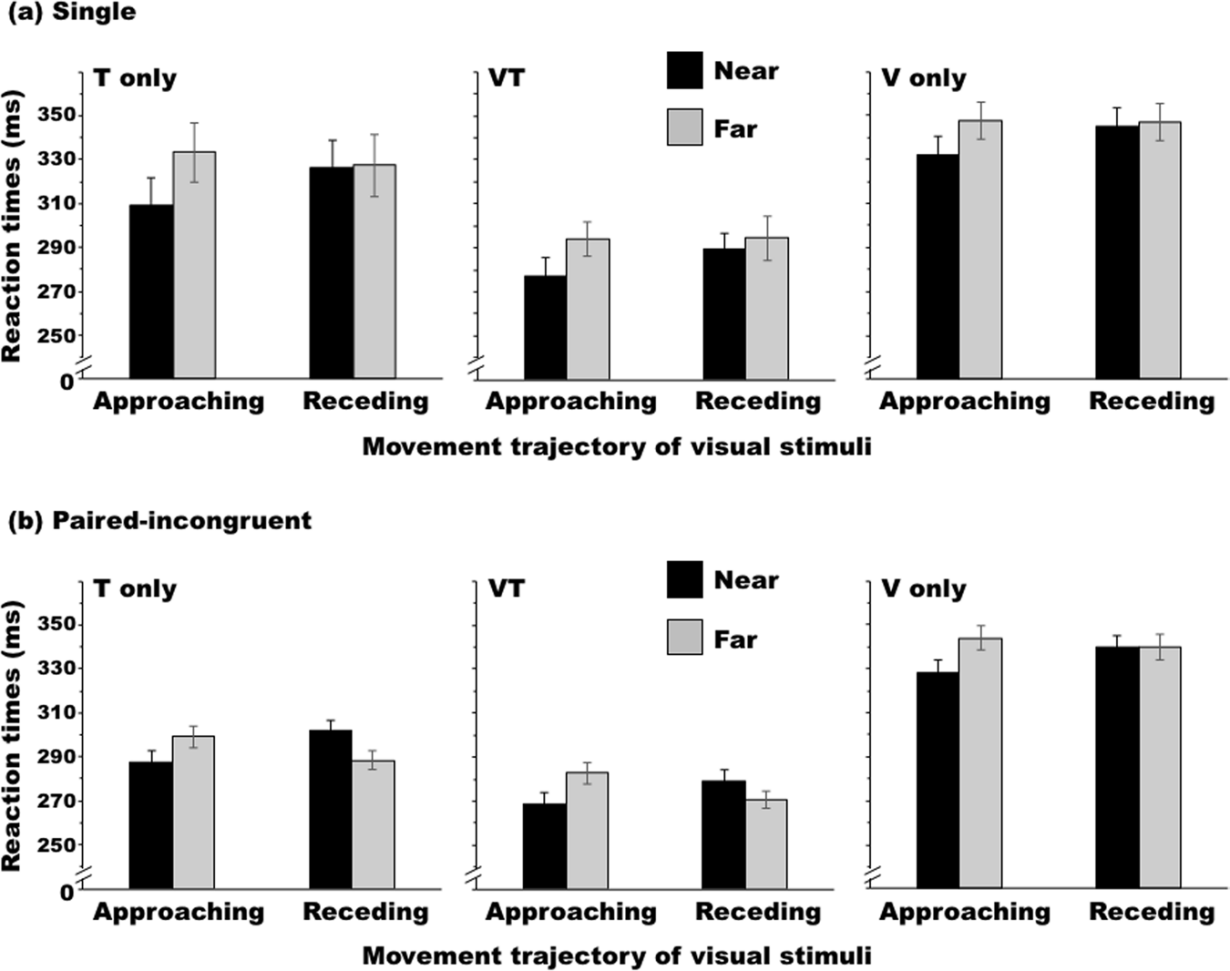
Average Experiment 3 reaction times (RTs) across the participants (n = 18) under the (**a**) single condition and (**b**) paired-incongruent condition. Error bars show standard errors of the means.

Figure 6 shows the results of Experiment 3. The error rates, including no-response errors, were 1.1 ± 1.4% for the single condition and 1.2 ± 2.2% for the paired-incongruent condition. I performed two-way ANOVAs in the same manner as those in the previous experiments. Under the single condition, the ANOVA revealed a significant interaction for all target types (T-only: *F*_1,17_ = 6.11, *p* = 0.024, *η*_*G*_^2^ = 0.011; VT: *F*_1,17_ = 5.13, *p* = 0.037, *η*_*G*_^2^ = 0.007; V-only: *F*_1,17_ = 8.68, *p* = 0.009, *η*_*G*_^2^ = 0.010). Further analysis of this interaction revealed that the RTs were shorter when the disk approached in the near space than when it approached in the far space (T-only: *F*_1,34_ = 20.32, *p* < 0.001, *η*_*G*_^2^ = 0.373; VT: *F*_1,34_ = 24.18, *p* < 0.001, *η*_*G*_^2^ = 0.476; V-only: *F*_1,34_ = 14.09, *p* < 0.001, *η*_*G*_^2^ = 0.465). Under the paired-incongruent condition, the ANOVA revealed a significant interaction for all target types (T-only: *F*_1,17_ = 19.79, *p* < 0.001, *η*_*G*_^2^ = 0.088; VT: *F*_1,17_ = 47.80, *p* < 0.001, *η*_*G*_^2^ = 0.081; V-only: *F*_1,17_ = 13.29, *p* = 0.002, *η*_*G*_^2^ = 0.027). Analyses of this interaction revealed that the RTs were shorter when the disk approached in the near space than when it approached in the far space (T-only: *F*_1,34_ = 11.46, *p* = 0.001, *η*_*G*_^2^ = 0.182; VT: *F*_1,34_ = 38.86, *p* < 0.001, *η*_*G*_^2^ = 0.355; V-only: *F*_1,34_ = 17.30, *p* < 0.001, *η*_*G*_^2^ = 0.470), but this relationship was reversed when the disk receded from the hand under the T-only and VT conditions (T-only: *F*_1,34_ = 16.52, *p* < 0.001, *η*_*G*_^2^ = 0.243; VT: *F*_1,34_ = 15.79, *p* < 0.001, *η*_*G*_^2^ = 0.183; V-only: *F*_1,34_ = 0.01, *p* = 0.980, *η*_*G*_^2^ < 0.001). Thus, under the paired-incongruent condition, the remapping effects were observed for the T-only and VT conditions, meaning that the current remapping effects did not occur in a body-part (hand) specific manner.

## Discussion

Recent neurophysiological and neuroimaging studies have shown that some neural populations in monkey parietal^16^ and human premotor cortices^17^, which are assumed to represent one’s own PPS, also respond to other’s PPS. In this behavioral study, I explored this phenomenon using a visuotactile interaction task. When the participants performed the task alone, they detected visual, tactile, and visuotactile targets sooner especially when approaching visual stimuli were located close to their hands rather than far from their hands. My novel findings were obtained under the paired condition, in which participants detected targets sooner when visual stimuli approached either their own or a partner’s hand. In other words, when the visual stimuli approached the partner’s hand, the participants behaved as if the partner’s near space was their own near space. Intriguingly, this did not require prior induction of enfacement illusions^30^. Experiment 2 revealed that the observed remapping effect occurred even when the participants were not acquainted with their partner and did not occur when rubber hands were presented instead of real ones. Furthermore, Experiment 3 showed that the remapping effect appeared even when the body parts to which visual stimuli were presented were different between the participant (hand) and partner (chest).

Many studies have suggested the existence of PPS representations in the human brain by showing that responses to visuotactile and audiotactile stimuli near the body are stronger than those far from the body. For example, visual stimuli presented close to the body improve tactile detection^39^, provide stronger facilitation of tactile discrimination^40^, and do more to promote tactile extinction in patients with right brain damage^41–43^. Further, some studies using approaching and receding auditory and visual stimuli have reported that the approaching stimuli provide stronger facilitation of tactile detection^29,44^ and stronger activation of parietal brain areas, which are assumed to process visuotactile stimuli^28^. Several other studies have shown that tactile, visual, and visuotactile processing are facilitated in the peri-hand space^45,46^. Thus, the facilitation of visual, tactile, and visuotactile target detection observed in this study under the single condition is strongly consistent with the findings of previous studies and provides further evidence for the existence of PPS representation in the human brain. It should be noted that there were inconsistencies between Experiments 1 and 2 in terms of receding condition results under the single condition. An effect of distance under the receding condition was observed in Experiment 1 but not in Experiment 2. The effect of distance for receding stimuli may somehow be unstable relative to the effect of distance for approaching stimuli. In fact, some electrophysiological studies have showed that some PPS-encoding visuotactile neurons in the ventral intraparietal area selectively respond to stimuli moving in certain directions, including approaching and receding visual stimuli^38^. Others studies have reported that body-approaching visual stimuli in the near space evoke greater activity in the visuotactile neurons than those in the far space^26^. Further studies are needed to clarify this point.

Experiment 3 revealed that the observed remapping effects occurred even when the body parts to which visual stimuli were presented were different between the participants (hand) and partner (chest). This indicates that the participants’ performance cannot fully be explained by the remapping of hand-centered PPS. There are two possible mechanisms to explain this. One possibility is that several body-part selective PPS representations are involved in this phenomenon. Because all body parts were closely located, the moving visual stimuli could to some extent activate both the hand-centered and chest/body stem centered PPS representations. Considering that Ishida *et al*.^16^ showed that PPS remapping in monkeys occurred in a body-part selective manner and Serino *et al*.^47^ showed body-part selectivity of human PPS, a more sophisticated experiment may be able to provide evidence that human PPS remapping also occurs in this manner. Another possibility is that the participants share body-derived attentional/arousal mechanisms with their partner. It is reported that approaching/looming stimuli facilitate tactile or orienting responses as compared to receding stimuli in humans^29,48,49^. A likely explanation for this bias is that responses to approaching/looming is essential for survival, so that attention to the body or arousal level of the whole body is increased for subsequent defensive behaviors and collision avoidance. Thus, sharing/remapping body-derived attentional/arousal mechanisms between the participants and partner could facilitate the participants’ responses especially to tactile stimuli (i.e., T and VT conditions) when visual stimuli approached the partner’s body in the partner’s near space. Humans shares representation with others at several information processing levels such as action^4,5,11^, emotion^50^, and perception^6–10,12,13^. Thus, it is likely that humans also share body-derived attentional/arousal mechanisms with others. These explanations are not exclusive and could equally contribute to the current results.

In contrast with Maister *et al*.^18^, I observed strong remapping effects without the induction of body ownership illusions. This difference might arise from the fact that Maister *et al*.^18^ used only stimuli approaching the participant, while I used both approaching and receding stimuli. This is important because the remapping effects in my experiments imply that objects receding from the participants are approaching the partner and hence are equivalent to objects approaching the participants. Alternatively, task-relevancy of moving objects might be involved, as I used visual-only, visuotactile, and tactile-only conditions. My participants would have paid more attention to moving stimuli than participants in previous studies did, which might have promoted the observed effects. Another difference between the present and previous studies is that, in this study, the partner responded to the targets. In the preliminary observations, I found that results under which the partner responded were more stable than those under which the partner did not respond, so I used the latter only. It could be that the involvements of another person in the task induces strong and stable utilization of one’s own PPS representation for mapping of another’s PPS or remapping body-derived attentional/arousal mechanisms. Thus, my experimental paradigm may be ideally suited to eliciting remapping effects.

Interestingly, in contrast to studies showing that mere exposure to dummy hands can influence one’s own positional senses and PPS representation^28,36^, the results of Experiment 2 showed that dummy hands did not induce PPS remapping. This is consistent with the results of the previous neurophysiological and neuroimaging remapping studies^16,17^. Behavioral and neuroimaging studies have reported that the mirror system has a human bias, meaning that this system is more strongly activated by observing human actions than by observing non-biological movements^51^. Thus, this type of remapping might require something other than simply seeing another individual’s body part, such as an assumption that the other individual can behave just like oneself.

In conclusion, my findings suggest that the human brain has some mechanisms for connecting the PPS and/or body of others with one’s own. However, it remains unknown if the visuotactile neurons and areas in the parietal^16^ and premotor cortices^17^ are involved in the present findings because body-part selectivity was not observed. Nevertheless, the remapping effect observed in the present study could also be useful for understanding the actions^1–3^ and tactile perceptions^6–10^ of others and may contribute to optimizing one’s behaviors in protecting another person or oneself from threats.

## Methods

### Participants

For Experiment 1, ten undergraduate students with normal or corrected-to-normal visual and tactile acuity were recruited (four women, mean age: 21.8 ± 0.42 [standard deviation] years). A 22-year-old man who knew the purpose of the experiment was also recruited to serve as the participants’ partner in trials requiring one. The participants became acquainted with the partner before the experiments. For Experiment 2, 11 different students (seven women, mean age: 18.8 ± 0.69 years) were recruited as participants and a 23-year-old man who had never met the participants was recruited to serve as their partner. For Experiment 3, 18 students (10 women, mean age: 21.4 ± 1.33 years; 3 woman also participated in Experiment 2) were recruited as participants and a 50-year-old woman was recruited to serve as their partner. Half of the participants first met with the partner. Informed consent was obtained from all participants and partners. The participants were unaware of the experiment’s purpose. The study design was approved by the Ethics Committee of the Kumamoto University Graduate School of Social and Cultural Sciences, and the experiments were performed in accordance with the principles of the Declaration of Helsinki.

### Apparatus and stimuli

A schematic of the experimental setup is shown in Fig. 1. A vibrotactile stimulator with a 0.95-cm diameter (C1034; SHICOH, Yamato, Japan) was attached to the tip of the index finger. It was set to vibrate at a 300-Hz frequency, a 100-ms duration, and an amplitude far above the detection threshold. Visual stimuli were projected onto a screen on the table via a projector (NP-L50WJD; NEC, Tokyo, Japan). A red fixation point (16.9 cd/m^2^, 0.8 cm in diameter) was presented at the table’s center on a black background (1.0 cd/m^2^). The fixation point was located 17 cm from the tip of the index finger. A disk (98.7 cd/m^2^, 1.2 cm in diameter) approached or receded from the participant’s hand at 9 cm/s for 1 s in either the area beyond the fixation point (the far space) or the area nearer the participant (the near space). When the disk moved in the near space, the very end of the fingertip was where the approaching disk vanished and the receding disk appeared. When the disk moved in the far space, the corresponding point was 8 cm beyond the fixation point. The disk was initially white and turned green in some trials (see the *Procedur*e section for details). The experiment was controlled with an IBM-compatible computer (NEXTGEAR; MouseComputer, Tokyo, Japan) running 64-bit Windows 7 (Microsoft, Redmond, Washington). The experiment was controlled through MATLAB R2014b (MathWorks, Natick, MA) with the Psychophysics Toolbox extensions^52,53^. The participants stood in order to increase the visibility of the visual stimuli in the far space and touched their foreheads to an apparatus connected to the projector in order to keep the viewing distance constant. When the participants performed the task with the partner, the partner stood (Experiments 1 and 2) or sat (Experiment 3) across the table while facing the participants and touching his forehead to the opposite side of the apparatus. The tips of the participants’ and partner’s left index fingers were 34 cm apart for Experiments 1 and 2. In the rubber hand condition of Experiment 2, a dummy hand (14 cm long) was placed instead of the partner’s hand. In Experiment 3, the partner’s left arm hung vertically toward the floor. In all experiments, a vibrotactile stimulator was attached to the tip of the index finger of the partner. The experiment room was dimly lit, but the projector’s light illuminated the partner’s hand for the participants.

### Procedure

There were two moving trajectories for the disk (approaching and receding), two spaces where the disk was presented (near and far spaces), and three target types (T-only, V-only, and VT). Under the T-only condition, a vibrotactile stimulation was presented during the disk’s movement. Under the VT condition, both vibrotactile stimulation and a white-to-green-change in the moving disk’s color were presented during the disk’s movement. The disk was initially white and turned green. Under the V-only condition, only a change in the moving disk’s color was presented during the disk’s movement. Theoretically, only the T-only condition was demanded, but my pilot study showed that visual stimuli did not affect the detection of tactile stimuli when participants were asked to report only tactile stimuli, perhaps because the visual stimuli were task-irrelevant in this case. Thus, this study also included the V-only and VT conditions. The T-only, V-only, and VT conditions were each presented in 30% of trials, and the remaining 10% were catch trials featuring no target presentation. The target appeared 0.2, 0.5, or 0.8 s after the disk started moving. This means that the target appeared when the disk was 1.8, 4.5, or 7.2 cm from the tip of the finger under the near condition, or 26.8, 29.5, or 32.2 cm from it under the far condition. Once the disk’s color changed, it remained constant until the disk disappeared. The disk approached the hand in half the trials and receded from it in the others. The disk was likewise presented in the near space for half the trials and in the far space for the others. These trial types (target type × spatial distance × trajectory) were pseudo-randomly presented. The participants were asked to respond to the target as soon as possible by pressing a response button. In Experiment 1, the participants performed six experimental sessions, each consisting of 120 trials, and took short breaks between sessions. Half the participants performed the task under the single condition in the first three sessions and under the paired condition in the last three, while the other participants performed the task in the reversed order. Under the paired condition, the partner and the participants responded to the same stimuli. In Experiment 2, in addition to the single and paired conditions, there were a rubber hand condition under which, instead of the partner and his real hand, a left forearm rubber hand with a vibrator attached to the tip of the index finger was placed on the table. The single, paired, and rubber hand conditions were presented in pseudo-random order. Experiment 3 included single and paired-incongruent conditions. The single condition was the same as in previous experiments. In the paired-incongruent condition, the body parts from which visual stimuli approached were the hand for the participants and the center of the chest for the partner. For both Experiments 2 and 3, the participants performed two experimental sessions (each consisting of 120 trials) for each condition. Before the experimental sessions, each participant completed one practice session of approximately 20 trials to ensure understanding of the task. The participants were also instructed to focus on the fixation point throughout the experimental session and not look directly at their hands or the partner’s hand, though no gaze monitoring system was used. No feedback was provided to the participants.

### Statistical analyses

Trials in which the RTs exceeded ± 2 standard deviations or the lower limit (150 ms) were discarded from the following analyses as errors. I applied two-way repeated-measures ANOVAs with the within-participant factors of target distance (near or far) and trajectory (approaching or receding) for each target type (T-only, VT, or V-only) under the single and paired conditions in Experiment 1, under the single, paired, and rubber hand conditions in Experiment 2, and under the single and paired-incongruent conditions in Experiment 3. For interactions identified as significant (*p* < 0.05) or nearly significant (0.05 ≤ *p* < 0.10) in the ANOVA, I tested simple main effects.

### Data Availability

The datasets generated and analyzed during the current study are available from the corresponding author on reasonable request.

## Electronic supplementary material

Supplemental Figure S1
