# Supplementary material for: A behavioral approach to shared mapping of peripersonal space between oneself and others
Source: Sci Rep. 2018 Apr 3;8:5432. doi: 10.1038/s41598-018-23815-3 (PMC5882808; doi:10.1038/s41598-018-23815-3)
Supplement: Supplementary file 1 — Supplemental Figure S1 [file 41598_2018_23815_MOESM1_ESM.pdf]

A behavioral approach to shared mapping of peripersonal space between oneself and others

Wataru Teramoto

Figure S1

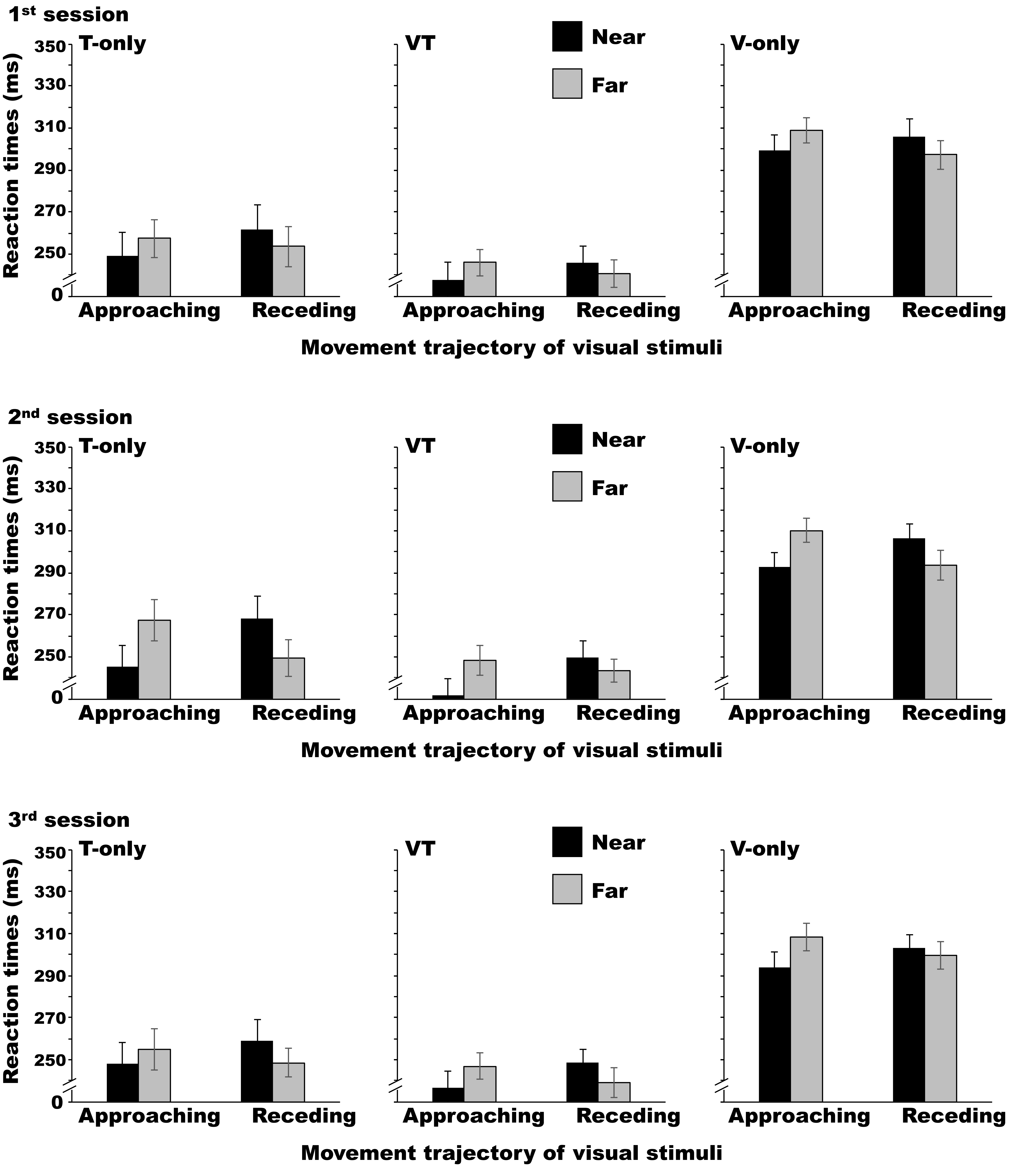

1st session
2nd session
3rd session
T-only
VT
V-only
Near
Far
Reaction times (ms)
350
330
310
290
270
250
0
Approaching
Receding
Movement trajectory of visual stimuli

Supplemental Figure S1. Average reaction times (RTs) across the participants (n = 10) in each session when performing the task with a partner (paired condition). I investigated whether the ongoing shared sensory experience between the participants and the partner contingently induced body ownership illusions such as enfacement illusions [35] and consequent remapping [18] because the participants and the partner responded to all stimuli together. The remapping effect should have been stronger in later sessions if body ownership illusions were involved. I conducted three-way repeated-measures analyses of variance (ANOVAs) with the within-participant factors of session (1, 2, or 3), target distance (near vs. far), and trajectory (approaching vs. receding) on the RT data for each target type. The ANOVAs revealed no significant main or interaction effects related to the session factor, but they did identify an interaction between distance and trajectory in all sessions ($Fs_{(1, 9)} > 13.59$, $ps < .005$, $\eta_G^2s > .067$). Further analysis of this interaction revealed that, in every session, the RTs were significantly shorter when the circle approached in the near space than when it approached in the far space ($Fs_{(1, 18)} > 9.67$, $ps < .006$, $\eta_G^2s > .257$), but this relationship was reversed for receding circles ($Fs_{(1, 18)} > 5.53$, $ps < .030$, $\eta_G^2s > .323$). Thus, the remapping effect occurred in all sessions and was not likely to increase in later sessions. Error bars show standard errors.
